# Using brain potentials to understand prism adaptation: the error-related negativity and the P300

**DOI:** 10.3389/fnhum.2015.00335

**Published:** 2015-06-12

**Authors:** Stephane J. MacLean, Cameron D. Hassall, Yoko Ishigami, Olav E. Krigolson, Gail A. Eskes

**Affiliations:** ^1^Cognitive Health and Recovery Research Lab, Departments of Psychiatry, and Psychology & Neuroscience, Brain Repair Centre, Life Sciences Research Institute, Dalhousie UniversityHalifax, NS, Canada; ^2^Neuroeconomics Lab, School of Exercise Science, Physical, and Health Education, University of VictoriaVictoria, BC, Canada

**Keywords:** prism adaptation, error-related negativity, P300, visuo-spatial neglect, feedback, event-related potentials, strategic recalibration, spatial realignment

## Abstract

Prism adaptation (PA) is both a perceptual-motor learning task as well as a promising rehabilitation tool for visuo-spatial neglect (VSN)—a spatial attention disorder often experienced after stroke resulting in slowed and/or inaccurate motor responses to contralesional targets. During PA, individuals are exposed to prism-induced shifts of the visual-field while performing a visuo-guided reaching task. After adaptation, with goggles removed, visuomotor responding is shifted to the opposite direction of that initially induced by the prisms. This visuomotor aftereffect has been used to study visuomotor learning and adaptation and has been applied clinically to reduce VSN severity by improving motor responding to stimuli in contralesional (usually left-sided) space. In order to optimize PA's use for VSN patients, it is important to elucidate the neural and cognitive processes that alter visuomotor function during PA. In the present study, healthy young adults underwent PA while event-related potentials (ERPs) were recorded at the termination of each reach (screen-touch), then binned according to accuracy (hit vs. miss) and phase of exposure block (early, middle, late). Results show that two ERP components were evoked by screen-touch: an error-related negativity (ERN), and a P300. The ERN was consistently evoked on miss trials during adaptation, while the P300 amplitude was largest during the early phase of adaptation for both hit and miss trials. This study provides evidence of two neural signals sensitive to visual feedback during PA that may sub-serve changes in visuomotor responding. Prior ERP research suggests that the ERN reflects an error processing system in medial-frontal cortex, while the P300 is suggested to reflect a system for context updating and learning. Future research is needed to elucidate the role of these ERP components in improving visuomotor responses among individuals with VSN.

## Introduction

Exposure to prism goggles that cause a lateral shift in the visual field produces an initial *direct effect* on aiming during visuomotor tasks such as goal-directed reaching. As a result, individuals make reaching errors in the direction of the goggle-induced visual shift. These errors are soon corrected with repeated practice, signaling *adaptation* to the visual shift. Finally, when the goggles are removed, there is a significant *after-effect* in reaching accuracy: healthy participants now make reaching errors in the opposite direction to the preceding prismatic shift. Interestingly, the aftereffect has been shown to improve some visuomotor and perceptual deficits experienced by individuals post-stroke experiencing a spatial attention disorder called visuo-spatial neglect (VSN) (Tilikete et al., 2001; Farnè et al., 2002; Angeli et al., 2004a,b; Keane et al., 2006; Rode et al., 2006; Serino et al., 2007, 2009; Jacquin-Courtois et al., 2008; Fortis et al., 2010; Shiraishi et al., 2010; Turton et al., 2010; Vangkilde and Habekost, 2010; Watanabe and Amimoto, 2010; Mizuno et al., 2011). VSN is a syndrome that often follows right hemisphere stroke, and less often left-hemisphere stroke. Persons showing VSN are typically characterized as having difficulty orienting and responding to stimuli in contralesional (i.e., typically left) space. Thus, a principal symptom is absent, slowed and/or inaccurate motor responses to contralesional stimuli. Neglect patients experience problems with daily activities such as eating, grooming and mobility. There is substantial evidence that when left VSN patients are exposed to a PA task, the leftward shifting after-effect provides benefits to their scanning and reaching performance (Rossetti et al., 1998; Frassinetti et al., 2002; Keane et al., 2006; Serino et al., 2006; Striemer and Danckert, 2007; Nys et al., 2008a; Sarri et al., 2008; Schindler et al., 2009; Bultitude and Rafal, 2010; Yang et al., 2013) although benefits are not always seen (McIntosh et al., 2002; Dijkerman et al., 2003; Morris et al., 2004; Datié et al., 2006; Humphreys et al., 2006; Rousseaux et al., 2006; Nijboer et al., 2008; Nys et al., 2008b; Turton et al., 2010; Sarri et al., 2011).

While visuomotor accuracy may improve after PA, there remains a limited understanding of what neuro-cognitive processes underlie the improvements, or how these neuro-cognitive processes change as a result of PA. This limitation may serve as a barrier to developing better procedures and proper translation of PA to clinical settings. Indeed, a major review of translational gaps regarding PA (Barrett et al., 2012) suggests a need for neuroscience-based methods to both categorize and target neglect symptoms using prism adaptation. A critical step in advancing this knowledge is to investigate neural processes that underlie changes in visuomotor responses during PA, first among healthy adults. This can enable us to understand the impact PA has on those processes, and thus refine the technique for its clinical application among neglect patients.

A prominent theory explaining cognitive mechanisms responsible for PA among healthy adults is described by Redding and Wallace (1997, 2002, 2006) and Redding et al. (2005). Here, they suggest that two main processes are engaged which permit people to both adapt to the prismatic visual displacement and also experience visuomotor aftereffects. Redding and Wallace first describe a process of *strategic recalibration*: a high-level form of general motor learning where subjects amend their motor program in response to performance errors and failure at achieving their motor goal. Second, they propose a process of *spatial realignment*: a low level, unconscious process where subjects undergo an internal remapping of sensory coordinates and reference frames (e.g., hand-head-eye) to accommodate the sensory discrepancy induced by prism goggles (e.g., between visual and proprioceptive information).

Strategic recalibration is most prominent during the early phase of adaptation in response to initial gross accuracy errors, whereas spatial realignment processes persist well after performance errors have been corrected during continued exposure to prism goggles. For example, Redding and Wallace (1993) showed that while compensation for direct effects may occur rapidly (e.g., within 30 trials), the magnitude of aftereffect continues to increase beyond those trials, such that aftereffects may be larger after 40, 50, and 60 trials of exposure. There is also evidence that the magnitude of PA aftereffect is a consequence of spatial realignment, and that under some circumstances strategic recalibration can reduce aftereffects, although it still contributes to the adaptation. For example, increasing visual feedback of the limb during pointing trajectory (e.g., making limb visible at the beginning of the reaching movement) enhances the participants' ability to deliberately recalibrate their motor trajectory and reduce accuracy errors. Under these conditions, the magnitude of after-effect is poor (Redding and Wallace, 1996; Herlihey et al., 2012). In contrast, only allowing visual feedback of the limb at the very end of the reach (limiting ability to recalibrate, but allowing realignment) increases the magnitude of after-effects.

In addition to the purported mechanisms for strategic control and spatial realignment, a number of neuroimaging studies have investigated regions of activation in the brain during the performance of visuomotor tasks under conditions of prism exposure. In a series of fMRI studies, Chapman et al. (2010), Luauté et al. (2009) and Danckert et al. (2008), implicated the importance of a cerebellar-parietal network over the course of adaptation, and showed the cerebellum to have increased activation during early trials—suggesting it has a role in strategic recalibration. Danckert, however, also showed early parietal activation. Küper et al. (2014) reported ventro-caudal dentate activation within the cerebellum during early trials, while cerebellar lobule activation during the later trials, suggesting a role in spatial realignment, has been reported by Donchin et al. (2012).

A region of interest outside of this network that has also garnered attention is the anterior cingulate cortex (ACC). Using fMRI, Danckert et al. (2008) reported increased activity in the ACC during the first three trials of prism exposure blocks compared to the last three. Interestingly, errors were only seen over those first three trials while the latter three were performed accurately. This result is in line with previous work providing strong evidence that ACC is associated with performance monitoring and error evaluation (Elliott and Dolan, 1998; Holroyd and Coles, 2002; Van Veen and Carter, 2002a,b; Kerns et al., 2004). In fact, there is a growing body of evidence that suggests the ACC is part of a neural learning system within medial frontal cortex (MFC) responsible for optimization of response selection (Holroyd and Coles, 2002, 2008).

Studies using event-related brain potentials (ERP) suggest that the MFC is the source of the *error-related negativity* (ERN), an ERP component sensitive to the first indication that the outcome of a selected action is worse than predicted (Dehaene et al., 1994; Holroyd et al., 1998, 2004; Ullsperger and von Cramon, 2001; Gehring and Willoughby, 2002; Van Veen and Carter, 2002a,b; Debener et al., 2005). The ERN is well documented over various experimental tasks where participants evaluate the correctness or accuracy of their selected action. In particular, the ERN is well documented over the course of tasks requiring visuomotor accuracy. For example, the ERN has been evoked by errors during manual tracking tasks (Krigolson and Holroyd, 2006, 2007a,b; Krigolson et al., 2008), accuracy errors during a shooting task (Bediou et al., 2012), aiming errors to complete a visual angle (Anguera et al., 2009), and accuracy errors during a postural control experiment (Hassall et al., 2014). Given that PA is a visuomotor task requiring repeated correction of errors, these studies suggest that a neural learning system within MFC, as indexed by the ERN, may play a role in the adaptation process.

So far, work by Vocat et al. (2011) is the only study that used the ERP methodology to study the ERN during PA. In this study, participants reached toward a randomly located dot on a monitor over 12 trials for 10 consecutive blocks that alternated between normal visual conditions and conditions of rightward-displacing prism goggle exposure (10°). This enabled participants to adapt and then de-adapt to the prism goggles repeatedly over several blocks. Vocat et al. measured ERPs evoked by error responses and correct responses. From these ERPs, the authors calculated difference-waves between trials in which participants hit the target and trials in which participants missed the target by four magnitudes of deviation (edge, slight deviation, mild deviation, large deviation). Vocat et al. showed that an early ERN-like component, with negative polarity, maximal at electrode FCz, and peaking at 76 ms post-response, increased in amplitude concomitantly with the size of errors. No ERN was reported for “edge” trials, however there was a small ERN for “slight” deviations and larger ERNs for both “mild” and “large” deviations. Furthermore, they reported a later component at the same electrode site (FCz) with a positive polarity, peaking at 185 ms post-response-onset. This positive component, like the ERN, increased in amplitude concomitantly with error size. These results thus lend support to the idea that a performance monitoring and learning system in MFC is engaged during PA.

In an effort to extend Vocat et al's (2011) findings, we examined the ERN over longer exposure blocks. As previously mentioned, Redding and Wallace (1993) showed that prolonged exposure to prism goggles contributes to increased magnitude of aftereffects even after correction for the initial direct effects occured. Longer exposure blocks thus allow us to not only examine neural signals during the adaptation process, but also examine whether there are changes in these signals over time during a course of adaptation that extends beyond the initial compensation period that corrects for the direct-effects. Furthermore, we designed the prism adaptation task so that participants would experience terminal exposure, i.e., the participants were able to view their limb only at the end of their reaching movement. This design isolates visual feedback to the point of touching the screen. Recent evidence (Làdavas et al., 2011) suggests that PA performed with terminal occlusion produces greater improvement of neglect based on the Behavioral Inattention Test, compared to PA performed with concurrent exposure. Given this design and the timing of visual feedback, we hypothesized that an ERN would be elicited at the moment participants terminated their reach and made contact with the screen.

In addition to the ERN, we extended Vocat et al's (2011) study further by also measuring an additional ERP: the P300. The P300 is a positive voltage deflection maximal at parietal-central electrode sites and peaks approximately 300–500 ms post-evoking-event. The P300 is suggested to signal either (1) a process of context-updating where information salient to participants' performance is added to their working model of the environment (Donchin and Coles, 1988, 1998), or (2) a phasic increase in noradrenergic firing in the locus-coeruleus (Nieuwenhuis et al., 2005) and implicated in facilitating the learning of responses to unexpected events (Yu and Dayan, 2005; Dayan and Yu, 2006).

For the present experiment, subjects performed goal-directed reaching toward targets on a touch-screen monitor over blocks of 45 trials. Like Vocat et al. (2011), subjects alternated between blocks of normal visual conditions and conditions of prism goggle exposure to enable repeated adaptation without effects carrying over from one prism block to the next. We averaged ERPs at the termination of reach (contact with monitor and presumed time of visual feedback), grouped according to trials in which subjects either hit or missed the target as we expected the miss trials would evoke the ERN compared to the hit trials. Furthermore, we binned both hits and misses according to three phases in each exposure block: early, middle, and late. The primary questions of interest were: (1) Are these error monitoring and learning signals associated with reaching outcomes during PA?; (2) If so, how would these signals change over time during the adaptation process? We hypothesized that, given Vocat et al.'s results, the ERN amplitude would significantly decrease from the early phase to both middle and late, as error size decreased. Similarly, we hypothesized that the P300 amplitude would also show a reduction between the early and late phases as motor learning proceeded and the subject became adjusted to reaching under the conditions of visual displacement.

## Materials and methods

### Participants

Twenty right-handed participants (7 male, 13 female) with no known neurological impairments and with normal or corrected-to-normal vision volunteered for the experiment. Participants were between the age of 19 and 34 years (mean age = 22.3 years, SD = 3.7). All participants provided informed consent in line with Dalhousie University's Policy on the Ethical Conduct of Research Involving Humans. The study was conducted in accordance with the ethical standards prescribed in the 1964 Declaration of Helsinki and subsequent amendments to the declaration.

### Apparatus

Participants performed goal-directed pointing movements toward a vertical-line target that appeared randomly in one of three target locations (middle, left, or right) on a 26 inch touch-screen monitor (Intellitouch Dual Monitor 2639L) placed on a desk 78 cm from the ground. Targets were 0.75 visual degrees wide and spanned the entire height of the monitor (32.5 cm). The middle target was located directly at the center of the screen's horizontal axis, while the midpoint of both the right and left targets were positioned 4 visual degrees, respectively, from the midpoint of the middle target.

A chinrest locked to the table was located 43 cm from the monitor and aligned with its horizontal center. Participants sat at an adjustable chair with their head on the chinrest, positioned such that their straight-ahead gaze would align with the center of the monitor. The height of the chair was adjusted for the participant to achieve optimal comfort with respect to the chinrest. Welder goggles were attached to the top of the chinrest, thus requiring participants to gaze through them in order to see the monitor. The type of goggles varied according to experiment block (see below).

A horizontal occlusion board prevented the participants from viewing their limb until the last 3 cm of the reaching movement trajectory. Finally, a keyboard used to time-lock experimental software and equipment to movement onset was positioned just beyond the chinrest with the spacebar aligned to the monitor's center.

The experiment was designed in Matlab (Mathworks, 2012) using the Psychophysics Toolbox. All visual stimuli and auditory cues were presented and timed appropriately using this toolbox. Matlab and Psychophysics Toolbox enable distinct EEG markers to be sent to a recording computer via an LPT port when particular events occur. Importantly, contact with the touch screen could simply be recorded analogously to a mouse-click. Furthermore, each pixel on the touchscreen corresponded to a specific X, Y value. Thus, the touch-screen could easily be implemented with Matlab and Psychophysics Toolbox to record behavioral data based on screen-touches (e.g., record timing of mouse-clicks, and record distance in pixels of screen-touch to target location), and also used to signal EEG markers.

### Procedure

Participants completed 13 blocks of 45 trials. Every block alternated between sham and prism goggles: clear goggles or goggles with prism patches (Insight Optometry Group, Halifax, Canada). For all participants, block one was conducted with sham goggles and used as a measure of baseline pointing accuracy. We varied the prism exposure condition according to two factors: direction (left and right) and degree of visual displacement (5.5° and 17°, or 15 and 30 if measured in diopters). This varying condition was used to prevent participants from anticipating the size or direction of displacement and gradually adapting more easily to each prism block over the course of the experiment. Consequently, participants were exposed to four different prism goggle conditions (prism-right 17°, prism-left 17°, prism-right 5.5°, prism-left 5.5°) in random order across a total of six prism blocks, each followed by a sham block. By alternating between sham and prism goggle exposure, participants would be able to adapt to prism goggles, then de-adapt with sham goggles, and then re-adapt to prism goggles in a continuous cycle from one block to the next. This alternation enabled repeated exposure to prism conditions without adaptation effects carrying over from previous blocks. Error responses were thus obtained throughout all blocks.

To initiate each trial, participants pressed and held the index finger of their pointing arm on the spacebar. After pressing the spacebar and holding for 500 ms a fixation-cross appeared for 500–700 ms, followed by the appearance of one of the three movement target positions in random order. Participants were instructed to continue to press the spacebar and not start their pointing movement until a short auditory cue (1000 Hz, 0.05 ms, 30 dB) was heard 800–1000 ms after the appearance of the target stimulus. After the tone, participants reached as “quickly and accurately as possible” to the presented target location on the monitor. Participants held their finger on their endpoint location (whether accurate or not) until the target disappeared (1000 ms post-touch). Target disappearance indicated the trial was complete and the participant could return to the spacebar in order to begin the next trial. They were not encouraged to take breaks between trials. If a participant released their finger from the monitor before the target disappeared, a “TOO FAST” message appeared on the monitor and the next trial began.

Participants were instructed to complete a fluid and ballistic movement on each trial. Furthermore, they were instructed to point high enough on the monitor to see their finger's location beyond the occlusion board upon making contact with the monitor. This was important to ensure participants received visual feedback regarding the accuracy of their pointing movement. To ensure understanding and compliance with the instructions, each participant performed a block of 10 practice trials toward a square target (1.75 cm^2^) located at the center of the monitor (positioned so that participants would be trained to point high enough to see their finger on each trial).

### Behavioral data collection

For each trial, size of pointing error and movement time (MT) were recorded. Error size was measured as the horizontal distance in visual degrees between the target location and participants' pointing location on the monitor. Movement time was recorded as the number of milliseconds between release of spacebar and contact with screen (termination of reach).

### Electroencephalography data collection

Electroencephalography (EEG) data were recorded from 64 electrode locations using Brain Vision Recorder software (Version 1.3) and Brain Vision PyCorder software (Brain Products, Munich, Germany). The electrodes were mounted in a fitted cap with a standard 10–20 layout and were recorded with an average reference. The vertical and horizontal electrooculograms (EOG) were recorded from electrodes placed above and below the right eye and on the outer canthi of the left and right eyes. Electrode impedances were kept below 20 kΩ. The EEG data collected with Brain Vision Recorder were sampled at 1000 Hz, amplified (Quick Amp, Brain Products), and filtered through a pass-band of 0.017–67.5 Hz (90 dB octave roll-off). The EEG data collected with Brain Vision Pycorder were sampled at 500 Hz, amplified (ActiCHamp, Brain Products), and filtered through a 8 kHz (−3 dB) anti-aliasing filter. No differences in data were observed between Brain Vision systems.

### Behavioral data analysis

The effects of goggle exposure type (baseline, prism, sham) and block phase (early, middle, late) on error size and MT were submitted to a 3 (exposure type) × 3 (phase) repeated measures ANOVA. Each phase was defined as *early*: trials 1–15, *middle*: trials 16–30, and *late*: trials 31–45. Mauchly's Test of Sphericity was applied to the data. If necessary, degrees of freedom were corrected by using the Greenhouse-Geisser estimate of sphericity. When applicable, a *post-hoc* multiple comparison test was conducted on main effects and interactions using the Sidak adjustment.

### Electroencephalography data analysis

The EEG data were re-sampled to 250 Hz, filtered offline through a 0.1–30 Hz pass-band, and 60 Hz notch phase shift free Butterworth filter, then re-referenced to the average of the two mastoid channels. The data were initially segmented into 800 ms epochs according to all event-markers (200 ms pre, 600 ms post). Ocular artifacts were corrected by applying the algorithm described by Gratton et al. (1983). All epochs were subsequently baseline corrected according to the 200 ms preceding the event's onset. Finally, we employed an artifact rejection that removed all trials in which a voltage change at any channel exceeded 10 uV per sampling point or the voltage change across the epoch was greater than 100 uV. In total, less than 10% of the data were discarded.

ERP components of interest belonged to epochs event-locked to the moment of contact with the screen—deemed to be the earliest indication of touch position accuracy relative to target. Here, segments were binned and averaged according to whether the trial was recorded as a “hit” or a “miss.” All reaches terminating within a 0.75° measure of accuracy were recorded as hits. Any reach outside 0.75° was recorded as a miss. Participants were simply instructed to hit the visible target as accurately as possible, and did not receive explanation of the precise criteria of a hit vs. a miss. Additionally, hit-epochs and miss-epochs were each averaged according to their respective phase (early, middle, late) determined by trial number.

In line with previous work (e.g., Krigolson et al., 2013, 2014), ERP components were measured based on visual inspection of grand-average waveforms (**Figures 3, 4, 7, 8**) and associated scalp topographies (**Figures 5, 9**). Visual inspection revealed that the peak difference between hits and misses (i.e., the ERN) occurred at fronto-central electrode site FCz. Measuring the ERN at electrode FCz is consistent with a substantial amount of literature (e.g., Krigolson and Holroyd, 2007a,b; Holroyd et al., 2009). The ERN was measured as the mean amplitude between 50 and 100 ms post-screen-touch. Given that the evoking stimulus is surmised to be the onset of visual feedback of reaching limb, and considering previous work (e.g., Miltner et al., 1997; Holroyd and Coles, 2002; Hassall et al., 2014; Krigolson et al., 2014), this latency appears slightly early. Nonetheless, the latency is consistent with many reports of the ERN (e.g., Gehring et al., 1993), although it is likely not evoked by an efference copy of the motor command—as hypothesized by Holroyd and Coles, but instead is evoked by onset of error information from seeing the reaching limb position. Visual inspection also revealed the P300 peaked at parieto-central electrode site Pz between 200 and 300 ms post-screen-touch—a slightly earlier peak latency than is often reported (e.g., Magliero et al., 1984). Pz is commonly reported as the electrode site with maximal activation from the P300 component (e.g., Krigolson and Holroyd, 2007a; Krigolson et al., 2008).

To examine the effects of phase, goggle exposure, and accuracy on ERPs, mean amplitudes between 50 and 100 ms and between 200 and 300 ms post-screen-touch were separately submitted to a 2 (accuracy: hit, miss) × 3 (phase: early, middle, late) × 2 (exposure: prism, sham) repeated measures analysis of variance (ANOVA). The baseline exposure condition was removed from the analysis due to insufficient trials to measure ERP data.

Finally, in an effort to replicate results found in Vocat et al. (2011), a separate analysis was conducted to measure the effect of error size on ERPs. It was necessary to measure the effect of error size averaged over phase and exposure, as including the additional factors would result in insufficient trials in each group to conduct a valid ERP analysis. Here, ERPs event-locked to contact with screen were collapsed across prism and sham blocks, as well as all early, middle, and late phases. The ERPs were only averaged according to four bins: hits, small errors, medium errors, and big errors. Hits were measured in the same fashion as the first analysis: any contact with screen within 0.75 visual degrees from target. Small misses were categorized as any contact with screen between 0.751 and 1.5 visual degrees distance from target. Medium errors were categorized as contact with screen 1.51–2.25 visual degrees from target. And large errors were categorized as any contact with screen 2.251 visual degrees or more from target. Mean amplitudes between 50 and 100 ms (ERN) and between 200 and 300 ms (P300) post-screen-touch were separately submitted to a one-way repeated measures ANOVA with error size (hits, small misses, medium misses, large misses) as the only factor.

The assumption of sphericity was tested via Mauchly's Test of Sphericity. Greenhouse-Geisser corrected degrees of freedom were used as necessary. *Post-hoc*, a multiple comparison test was conducted on main effects and interactions using the Sidak adjustment. An alpha level of 0.05 was set as a threshold of significance for behavioral and ERP statistical tests.

## Results

### Behavioral results: error size

**Figure 1:**
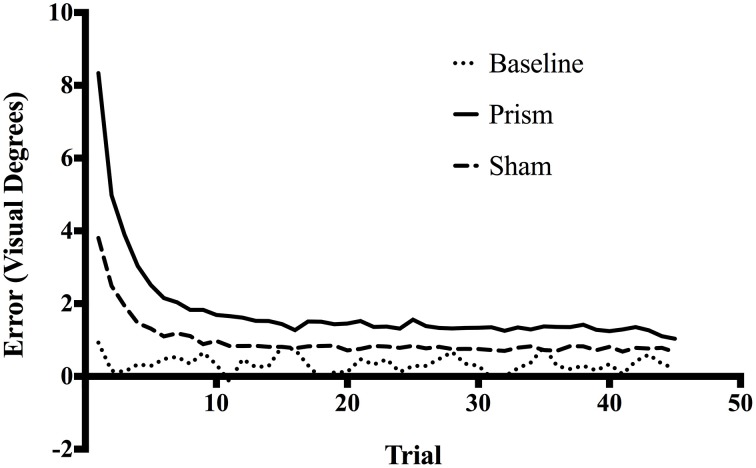
**Mean error size across all trials for Baseline, Prism, and Sham conditions**. All blocks had 45 trials, each corresponding to one reach toward a target. Error size corresponds to the horizontal distance in visual degrees between target location and final pointing location on screen.

Figure 1 presents the average trial-by-trial error size across all trials for each exposure type. A linear regression was calculated to predict error size based on trial number during the early phase of the experiment (i.e., only the first 15 trials of the experiment). For Baseline exposure, the analysis produced a non-significant regression equation, *F*_(1, 298)_ = 1.63, *p* = 0.20, *R*^2^ = 0.005, and a non-significant slope of -0.011 (*p* = 0.20). For Sham exposure, the analysis produced a significant regression equation, *F*_(1, 1798)_ = 519.5, *p* < 0.001, *R*^2^ = 0.22, and a significant slope of -0.15 (*p* < 0.001). For Prism exposure, the analysis also produced a significant regression equation, *F*_(1, 1798)_ = 469.4, *p* < 0.001, *R*^2^ = 0.2, and a significant slope of -0.32 (*p* < 0.001). As illustrated in Figure 1, these results suggest participants significantly improved accuracy at the task over the first 15 trials during both the Sham and Prism conditions, however they did not significantly change accuracy over the early course of the Baseline condition. See Figure S1 for trial-by-trial error size for each prism exposure condition separately (direction and degree).

**Figure 2:**
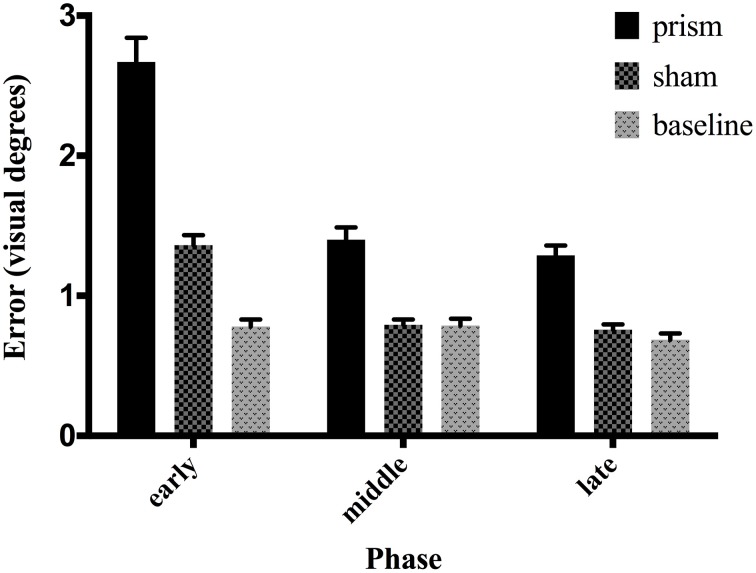
**Effect of phase and exposure condition on error size**. Each block was divided *post-hoc* into three phases: early (trials 1–15), middle (trials 16–30), and late (trials 31–45). The three exposure conditions correspond to baseline exposure (clear goggles), prism exposure (prism goggles) and sham exposure (clear goggles, but following prism adaptation). Error bars indicate standard error of the mean.

The main effect of phase on accuracy was significant, *F*_(1.16, 22.08)_ = 101.25, *p* < 0.05, partial η ^2^ = 0.84, as well as the main effect of exposure type *F*_(1.15, 21.91)_ = 114.34, *p* < 0.05, partial η^2^ = 0.85. There was also a significant interaction between phase and exposure, *F*_(1.6,30.52)_ = 68.04, *p* < 0.05, partial η ^2^ = 0.78 (see Figure 2). *Post-hoc* analysis revealed that accuracy did not differ across any phases in the baseline condition. In contrast, error size decreased across all three phases of the prism condition, from early to late, indicating adaptation was taking place. Finally, error size decreased in the sham condition from the early phase to both middle and late, but not between the latter two, showing initial after-effects followed by de-adaptation. See Table 1 for condition means and variance.

**Table 1:** **Mean error (in visual degrees) for all within-subject conditions (exposure, phase)**.

**Exposure**	**Phase**	**Mean**	**SD**	**95% CI**
Baseline	Early	0.78	0.22	[0.67, 0.88]
	Middle	0.78	0.21	[0.68, 0.88]
	Late	0.68	0.19	[0.59, 0.77]
Prism	Early	2.67	0.77	[2.31, 3.03]
	Middle	1.40	0.39	[1.21, 1.58]
	Late	1.28	0.31	[1.14, 1.43]
Sham	Early	1.36	0.32	[1.21, 1.51]
	Middle	0.79	0.17	[0.71, 0.87]
	Late	0.75	0.16	[0.68, 0.83]

*SD, standard deviation; 95% CI [x, x], upper and lower limit of 95% confidence interval*.

### Behavioral results: movement time

The main effect of phase was significant, *F*_(1.2, 23.91)_ = 4.13, *p* < 0.05, partial η ^2^ = 0.18. *Post-hoc* analysis showed that MT was significantly faster in the late phase compared to early phase, while neither differed significantly from middle. The main effect of exposure was significant, *F*_(1.09, 20.72)_ = 4.61, *p* < 0.05, partial η ^2^ = 0.19. *Post-hoc* analysis indicated that MT was faster in the sham condition compared to prism, while neither differed significantly from baseline. There was no significant interaction between phase and exposure, *F*_(1.63, 30.99)_ = 2.62, *p* = 0.09, partial η ^2^ = 0.12. See Table 2 for means and variance.

**Table 2:** **Mean movement time (seconds) for all within-subject conditions (exposure X phase)**.

**Exposure**	**Phase**	**Mean**	**SD**	**95% CI**
Baseline	Early	0.310	0.146	[0.242, 0.379]
	Middle	0.275	0.115	[0.221, 0.329]
	Late	0.277	0.122	[0.219, 0.334]
Prism	Early	0.272	0.109	[0.220, 0.323]
	Middle	0.267	0.112	[0.214, 0.319]
	Late	0.273	0.110	[0.221, 0.324]
Sham	Early	0.262	0.110	[0.211, 0.314]
	Middle	0.252	0.107	[0.202, 0.302]
	Late	0.248	0.104	[0.199, 0.297]

*SD, standard deviation; 95% CI [x, x], upper and lower limit of 95% confidence interval*.

In addition to this analysis, the effect of accuracy (hit vs. miss) on MT was measured using three separate paired sample *t*-test (one for each exposure conditions). This revealed that there was no difference in MT between hit-trials and miss-trials for any of the three exposure conditions; BL: mean difference (Mdiff) = 0.008 s, *t*_(19)_ = 0.57, *p* = 0.57; Prism: Mdiff = 0.004 s, *t*_(19)_ = 1.56, *p* = 0.16; Sham: Mdiff = 0.0007 s, *t*_(19)_ = 0.34, *p* = 0.74.

### Electroencephalographic results: the error related negativity

**Figure 3:**
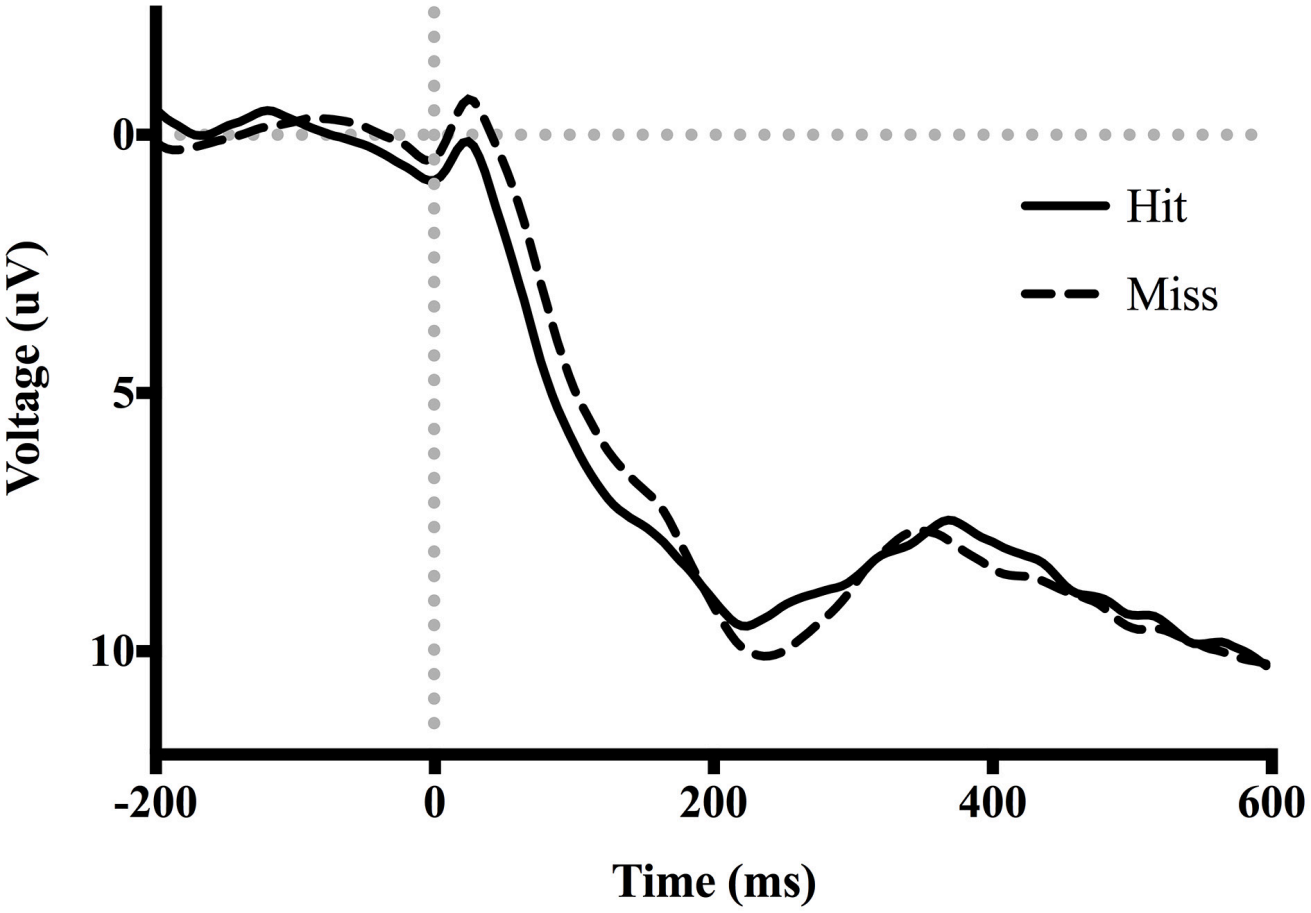
**Grand average ERP waveforms time-locked to screen-touch**. Between 50 and 100 ms an ERN is present.

**Figure 4:**
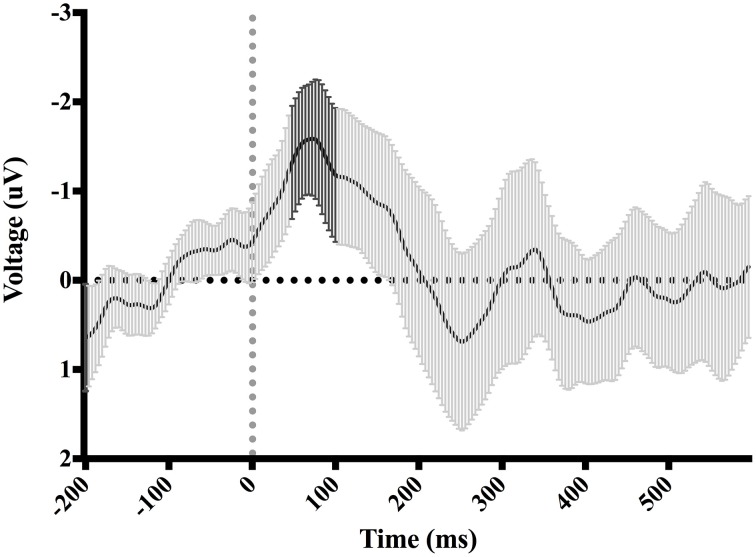
**Difference-waveform created by subtracting mean “hit” waveform from mean “miss” waveform**. Each time-point has a corresponding 95% CI. The bolded CIs reflect the time-window surrounding the peak negative amplitude used to measure the ERN.

**Figure 5:**
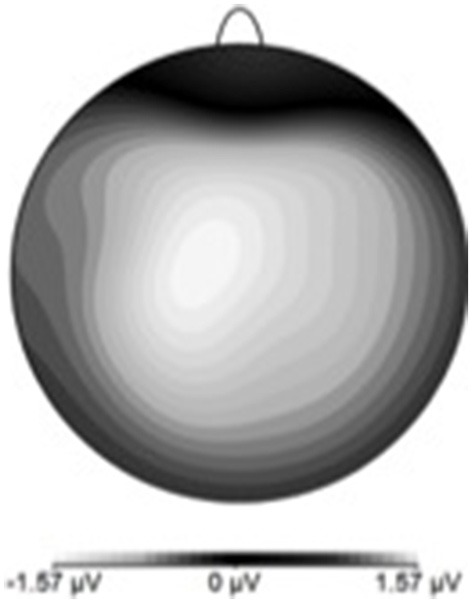
**ERN scalp topography derived by subtracting hit-ERP data from the miss-ERP data**. Negativity maximal at front-central electrode sites.

**Figure 6:**
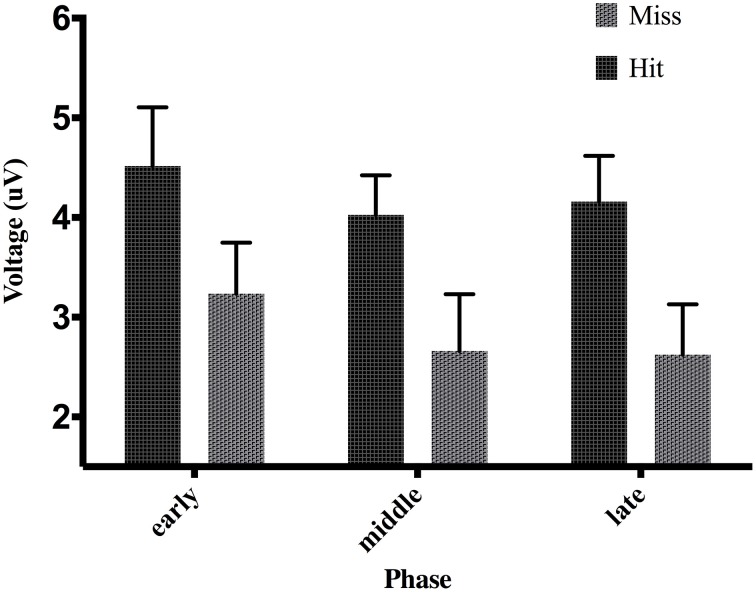
**Effect of phase and accuracy on mean voltage 50–100 ms post-screen-touch at electrode FCz**. Error bars indicate standard error of the mean.

Table 3 shows the proportion of hit trials compared to miss trials as a function of phase and exposure. For Table 3, “Near Hits” include trials in which participants came within 0.75 visual degrees of hitting the target on either side but did not hit the target directly. Figure 3 shows waveforms event-locked to screen-touch for both hits and misses. In order to determine the onset and peak of the ERN, we produced a difference-waveform by subtracting the mean “hit” voltage from the mean “miss” voltage across the screen-touch segment (−200 to 600 ms). We also measured 95% confidence intervals at each time point of the difference-wave to determine where the waveform showed significantly more negative amplitudes than zero (Figure 4). This revealed a negative component at electrode FCz (see Figure 5 for topography) peaking at approximately 75 ms post-screen-touch. Therefore, we submitted the mean amplitude 50–100 ms post-screen-touch to the ANOVA. This analysis revealed a main effect of accuracy (comparing hits against misses), *F*_(1, 19)_ = 20.9, *p* < 0.01, partial η^2^ = 0.52, and demonstrated that *miss* trials (mean = 2.84, SD = 3.33, SEM = 0.64, 95% CI [1.48, 4.19]) had a more negative voltage relative to *hit* trials (mean = 4.23, SD = 3.07, SEM = 0.56, 95% CI [3.05, 5.41]) (Figure 6). In other words, the difference between miss and hit trials affirmed inaccurate reaches had evoked an ERN-like component, but at an earlier latency than predicted based on previous work. The ERN amplitude was not affected by phase, *F*_(2, 38)_ = 1.48, *p* = 0.24, partial η^2^ = 0.07, exposure type, *F*_(1, 19)_ = 2.07, *p* = 0.16, partial η^2^ = 0.09, nor were there interactions between any of these measures (p's > 0.05).

**Table 3:** **Proportion of hits and misses binned according to Phase and Exposure conditions**.

**Exposure**	**Phase**	**Hits (%)**	**Near hits (%)**	**Misses (%)**
Baseline	Early	56	31	13
	Middle	56	31	13
	Late	62	29	9
Prism	Early	26	22	52
	Middle	36	28	36
	Late	40	27	33
Sham	Early	40	28	32
	Middle	57	30	13
	Late	58	31	11

A one-way ANOVA investigating the effect of error size on ERN showed that mean amplitude became more negative as accuracy decreased from hits to large errors (Table 4), *F*_(1.32, 25.12)_ = 5.42, *p* < 0.05, partial η^2^ = 0.22. However, *post-hoc* tests revealed that not all means were significantly different. Here, medium misses were significantly more negative than both hits and small misses. No other comparison reached significance. To further elucidate the effect of error size on ERN, a within-subject contrast analysis was also performed. Here, results showed a significant linear trend, *F*_(1, 19)_ = 8.23, *p* < 0.05, partial η^2^ = 0.3, indicating ERN amplitude increased as a function of increase in error size.

**Table 4:** **Mean ERP amplitude 50–100 ms post-screen-touch at electrode FCz, binned according to trials in which contact with screen corresponded to a hit, a small miss, a medium miss, and a large miss**.

**Error size**	**Mean**	**SD**	**95% CI**
Hit	3.36	2.06	[2.39, 4.33]
Small error	2.95	2.68	[1.69, 4.21]
Medium error	1.20	3.12	[-0.25, 2.67]
Large error	0.98	4.52	[-1.13, 3.10]

*SD, standard deviation; 95% CI [x, x], upper and lower limit of 95% confidence interval*.

### Electroencephalographic results: the P300

**Figure 7:**
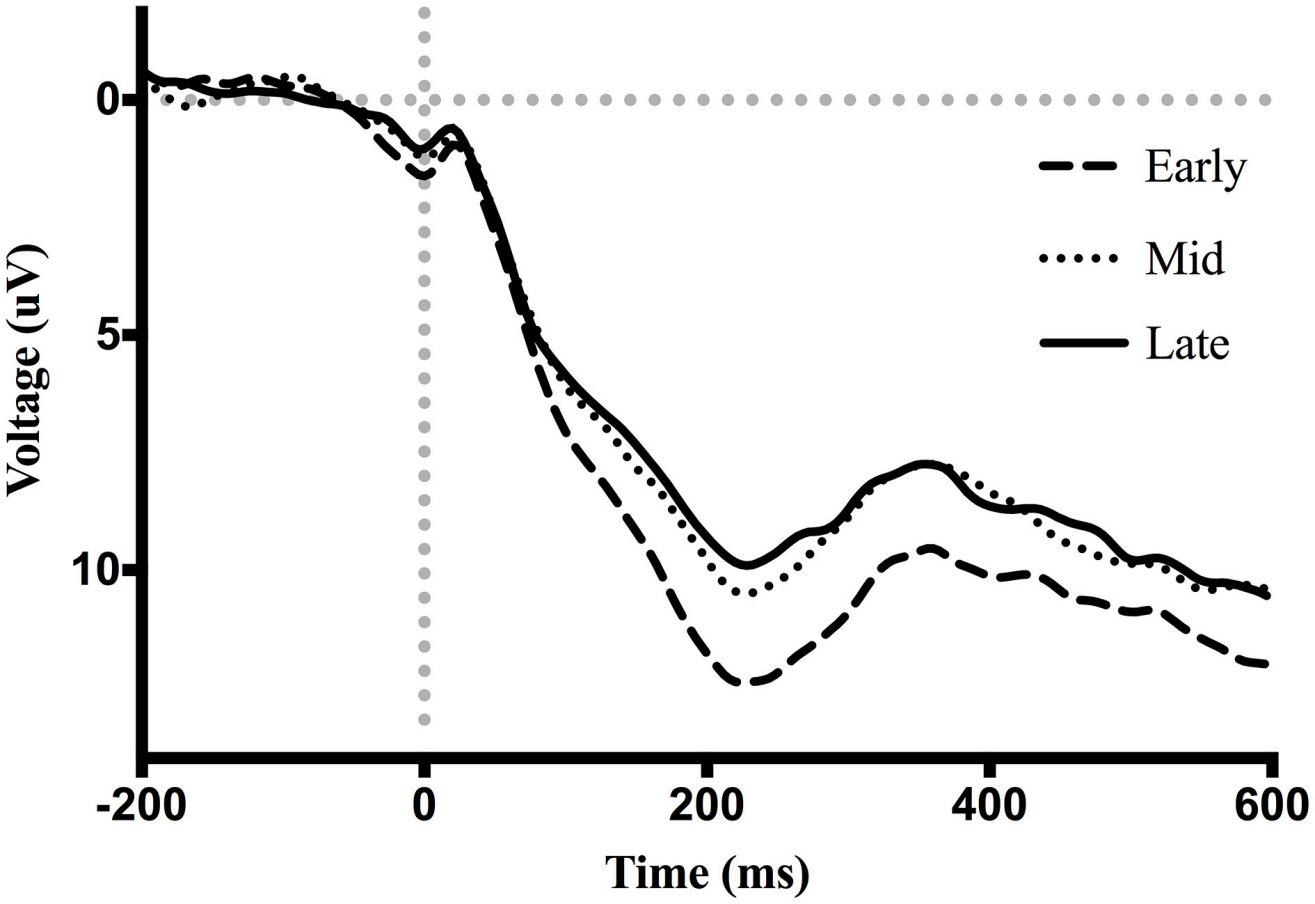
**Grand average ERP waveforms event-locked to screen-touch**. Between 200 and 300 ms a P300 is present with increased amplitude during the early phase.

**Figure 8:**
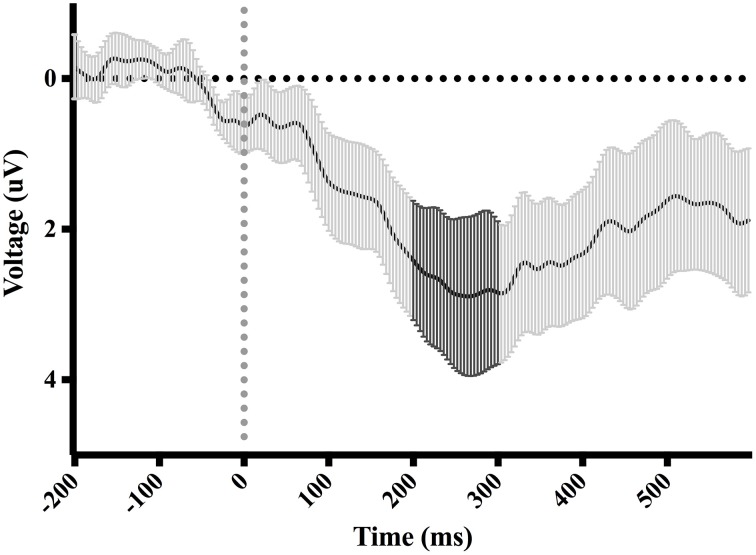
**Difference-waveform created by subtracting mean middle and late phase waveform from mean early phase waveform**. Each time-point has a corresponding 95% CI. The bolded CIs reflect the time-window surrounding the peak positive amplitude used to measure the P300.

**Figure 9:**
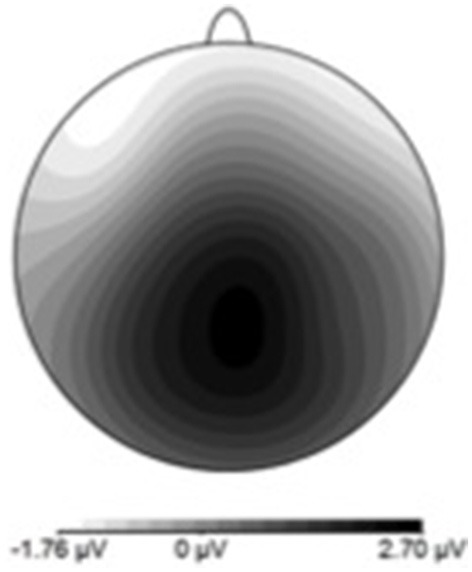
**P300 scalp topography derived by subtracting late-phase ERP data from the early-phase ERP data**. Positivity maximal at parieto-central electrode site.

Figure 7 shows waveforms event-locked to screen-touch, averaged according to the three phases of adaptation blocks: early, middle, late. Because we hypothesized the P300 would be sensitive to learning over the course of the blocks, we first measured its amplitude according to phase rather than accuracy. Visual inspection revealed a more positive P300 amplitude during early trials. To further elucidate this effect of phase, we created a difference-waveform (Figure 8) by subtracting the mean amplitude between middle and late phases from the early phase (see Figure 9 for topography). We also measured 95% confidence intervals at each time point of this difference-wave to determine the onset and peak of the isolated P300 component. This revealed a significantly more positive amplitude that peaked at approximately 250 ms post-screen-touch, thus the P300 was measure between 200 and 300 ms.

**Figure 10:**
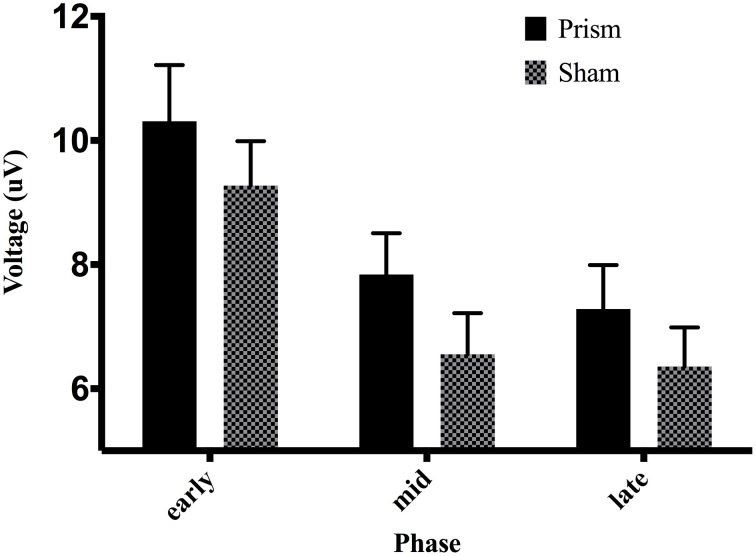
**Effect of phase and exposure on ERP voltage between 200 and 300 ms post-screen-touch at electrode Pz**. Error bars indicate standard error of the mean.

An analysis of the P300 revealed an effect of phase, *F*_(2, 38)_ = 27.04, *p* < 0.01, partial η^2^ = 0.58. *Post-hoc* decomposition revealed that the P300 was more positive during the early phase of adaptation (mean = 9.79, SD = 5.15, SEM = 1.05, 95% CI [7.58, 11.99]) compared to the middle (mean = 7.19, SD = 4.22, SEM = 0.87, 95% CI [5.37, 9.02]) and late phases (mean = 6.82, SD = 4.23, SEM = 0.88, 95% CI [4.97, 8.66]). However, no significant difference emerged between the middle and late phase. Our analysis of the P300 also revealed a main effect of exposure type, *F*_(1, 19)_ = 5.8, *p* < 0.5 partial η^2^ = 0.23, that demonstrated that P300 amplitude across all three phases during prism goggle adaptation (mean = 8.478, SD = 4.98, SEM = 0.64, 95% CI [1.48, 4.19]) was greater than P300 amplitude during sham goggle de-adaptation (mean = 7.395, SD = 4.41, SEM = 0.64, 95% CI [1.48, 4.19]) (Figure 10). P300 amplitude was not impacted by accuracy, *F*_(1, 19)_ = 0.07, *p* = 0.79, partial η^2^ = 0.004, nor were there any significant interactions (p's > 0.05).

While there was no effect of accuracy (hit vs. miss) on the P300, a one-way ANOVA did reveal a significant effect of error size on P300 amplitude [*F*_(1.84, 35.11)_ = 29.156, *p* < 0.001, partial η^2^ = 0.6]. *Post-hoc* tests indicate that only large errors produced significantly more positive amplitude compared to hits, small errors and medium errors. To further elucidate the effect of error size on P300, a within-subject contrast analysis was also performed. Here, results showed a significant linear trend, *F*_(1, 19)_ = 39.1, *p* < 0.001, partial η^2^ = 0.67, indicating P300 amplitude increased as a function of increase in error size. See Table 5 for means and variance.

**Table 5:** **Mean positive amplitude 200–300 ms post-screen-touch at electrode Pz, binned according to trials in which contact with screen corresponded to a hit, a small miss, a medium miss, and a large miss**.

**Error size**	**Mean**	**SD**	**95% CI**
Hit	6.86	3.86	[5.05, 8.67]
Small error	6.80	4.36	[4.76, 8.85]
Medium error	7.61	4.65	[5.43, 9.78]
Large error	10.48	4.68	[8.28, 12.67]

*SD, standard deviation; 95% CI [x, x], upper and lower limit of 95% confidence interval*.

## Discussion

In an effort to better understand the underlying brain mechanisms during PA that affect visuomotor responding, this experiment identified neural events over the course of PA sensitive to visual feedback and examined how they changed over the course of prolonged exposure blocks. Electroencephalography was collected while participants performed goal-directed reaching toward vertical-line targets over the course of several blocks. It was hypothesized that onset of visual feedback of accuracy would evoke two ERPs: (1) an ERN on trials in which the target was missed, and (2) a P300 sensitive to adaptation as learning progressed. Adding to previous literature, blocks consisted of 45 trials and were divided into three phases—early, middle, and late. It was expected that size of errors would significantly decrease over the course of the three phases of prism adaptation blocks. Thus, furthermore, it was also hypothesized that both ERN and P300 components would diminish in amplitude concomitantly with a reduction in accuracy errors across the three phases of PA blocks.

While the behavioral results are consistent with prior studies involving PA and support our hypotheses, the ERPs yielded both some unpredicted, and also novel results. Error size during PA blocks significantly decreased after the early phase showing that improvement at the task and adaptation was taking place. Moreover, the magnitude of errors at the onset of sham blocks was significantly greater than baseline and thus provides evidence that PA blocks produced the expected after-effects. The paradigm did indeed evoke an ERN and P300 at the onset of visual feedback. However, the ERN did not diminish in amplitude across the three phases of adaptation blocks as hypothesized. This result is discussed below. Importantly, we also found evidence that the P300 is modulated by phase—thus becomes smaller as the task progresses. Therefore, the P300 may signal a component of the adaptive learning process and it is also discussed further below. It is noteworthy that both ERPs showed a linear increase in amplitude concomitant with size of errors. This result thus replicates ERN findings in Vocat et al. (2011) where it was shown that size of error during PA modulates ERN amplitude.

### The error-related negativity

Consistent with our hypothesis, an ERN was measured at electrode FCz, 50–100 ms after contact with the screen during trials in which a miss occurred when compared to trials in which a hit occurred. Visual inspection of the ERN difference-wave (Figure 4) suggests that onset of the ERN begins nearly instantaneously upon screen-touch. Given that the ERN should show a delay between its evoking stimulus and its onset, this result suggests that the negative amplitude measured on “miss” trials is likely not evoked specifically by the event of touching the screen but rather the evoking stimulus is view of the limb prior to screen-touch. It is also important to note that there was no difference in movement time between hit-trials and miss-trials. Thus, we are confident the difference in ERPs is a result of error processing, rather than a movement artifact belonging exclusively to either the hit trials or the miss trials.

The terminal exposure method used for adaptation might account for this early onset of the ERN. Although the analyzed EEG segments were event-locked to the touch of the screen, it is likely that each participant began processing feedback by a number of milliseconds prior to this event—as the participants could see their reaching fingertip immediately before it landed on the screen. The moment the reaching fingertip became visible during this window is potentially the exact onset of feedback of accuracy, and could therefore account for an early ERN when measured from the onset of contact with screen.

This latter interpretation of the ERN result may inform us about how the brain responds to visuomotor errors during PA. The ERN result suggests first of all that a purported adaptive learning system in MFC may be involved in prism adaptation. A prominent theory holds that MFC is home to a system that reinforces actions that result in better outcomes than predicted, and suppresses actions that result in worse outcomes than predicted (the RL-ERN theory; Holroyd and Coles, 2002, 2008). Accordingly, the ERN is said to correspond to a dopaminergic signal in dorsal ACC evoked by the earliest indication that an outcome was worse than predicted (Schultz et al., 1993, 1997; Montague et al., 1996; Holroyd et al., 2004). There are a substantial number of studies that have source localized the ERN to MFC, or more specifically anterior cingulate cortex (Dehaene et al., 1994; Holroyd et al., 1998; Miltner et al., 2003; Herrmann et al., 2004; Ladouceur et al., 2006, 2007). Thus, the rapid improvements in accuracy during PA blocks after the early phase may come as a result of learning in MFC and suppression of erroneous response selection via dopaminergic activity. It is therefore noteworthy that the evoking stimulus, i.e., the event indicating that the outcome of response is worse than predicted, is not the screen-touch, but perhaps rather the onset of view of reaching limb. The screen-touch enables reliable feedback of accuracy regarding the task goal—to hit the target. However, the present result suggests that subjects use ongoing predictive information about limb position to gauge outcome of the end-goal during PA. The availability of feedback based on viewing the limb suggests the error signal could be based on visuo-proprioceptive information and deserves further investigation.

Interestingly, ERN amplitude did not diminish concomitantly with behavioral improvement across phases of prism adaptation—contrary to our hypothesis. This is particularly odd given the result that the ERN, when collapsed across sham and prism blocks, showed a trend to increase linearly with error size—which decreased from early to late phases. To account for this, it is possible that subjects' “expectancy” of accuracy differed across each phase. There is evidence that the ERN amplitude is affected by participants' expectancy of error: unexpected errors (or low probability errors) yield larger ERN amplitudes than expected errors (or high probability errors) (Holroyd and Coles, 2002; Holroyd et al., 2003). Therefore, it is important to note that the probability of committing a reaching error became smaller from the early phase to the late phase. For example, during PA blocks, the percentage of hits in the early phase is 26% (see Table 4), translating to roughly a 74% chance of missing the target. Conversely, the percentage of hits in the late phase is 40%, translating to roughly a 60% chance of missing the target. As a result, participants' expectancy of committing an error likely changed across phases. While errors may have been highly expected in the early phase, they were less expected in the latter phases. Thus, the unchanging ERN amplitude across phase may be explained by a combination of error size and error expectancy. Errors were larger in the early phase of PA blocks (thus increasing ERN amplitude), however, they were also more expected (thus decreasing ERN amplitude). The opposite effects would take place in the middle and late phase. Indeed, it is worth noting evidence that error frequency can impact error-related processes other than ERPs. For example, results from Notebaert et al. (2009) showed that post-error slowing was produced by an orienting response to infrequent errors, but was not produced by frequent errors. Post-response slowing was thus also observed following infrequent correct response (i.e., post-correct slowing).

### The P300

In addition to the ERN, it appears a second neural system proposed to reflect a process of context-updating and LC-NE phasic activity also functions in some capacity to engage changes in visuomotor responses during PA. A P300 response appeared sensitive to learning (independent of touch accuracy) by comparing all screen-touches (hit and miss) across three phases of PA—early, middle, late. During the early phase, a significantly increased positive voltage maximal at electrode Pz was measured between a latency of 200–300 ms after contact with screen.

The P300 response significantly declined during middle and late phases, concurrent with the improvements in accuracy and MT. The neural system responsible for the P300 might therefore subserve improvements at the PA task and taper its activity once improvements are achieved. The additional result that P300 amplitude tended to increase as a function of error size also supports this theory.

Current theories on the P300 component support the notion that this system could have a role in early adaptation. The context-updating theory, put forward by Donchin and Coles (1988), proposes that the P300 reflects a neural process of revising one's internal model of the environment. Such as the case, a P300 response is observed following stimuli/responses that require significant revision be made to the current model (Duncan-Johnson and Donchin, 1977; Pritchard, 1981). The P300 observed here may thus reflect a revision process in regards to participants' model of the experimental environment at the onset of PA blocks. Given the magnitude of errors during the early phase of PA blocks compared to baseline, participants clearly experience incongruencies regarding their expected performance and actual outcome. These incongruencies may be interpreted as discrepancies between expected environmental parameters and actual experienced environmental parameters. Exposure to the prismatic visual displacement would indeed require a revision to one's model of how the environment works—where intended motor commands no longer correspond to visual feedback of motor trajectory. Interestingly, although a contrast analysis suggested the P300 increased linearly with error size, amplitude did not significantly differ between hits and misses. The result that P300 amplitude was not strongly affected by accuracy would suggest that, if the P300 does indeed signal a process of context-updating, that the phenomena was evoked primarily by the visual displacement, but not necessarily only by errors resulting from the visual displacement.

The LC-NE theory of P300 (Nieuwenhuis et al., 2005; Nieuwenhuis, 2011) also carries interesting implications. According to this theory, the P300 reflects changes in noradrenergic firing in the locus coeruleus. The LC-NE projects to neocortex and hippocampal regions and has been shown to modulate responsivity of its target neurons (Berridge and Waterhouse, 2003), which can thereby impact efficiency of behavioral responses (Servan-Schreiber et al., 1990; Aston-Jones and Cohen, 2005). Studies on monkeys provide evidence that increased LC-NE activity increases exploratory behavior (Aston-Jones et al., 1994, 1997; Usher et al., 1999). In fact, a recent study with human subjects showed that P300 amplitude positively correlated with exploratory decision-making (Hassall et al., 2013). In our present study, the P300 may thus reflect increased LC-NE activity in response to participants' need to amend their current response strategy. While participants perform the visuomotor task with success during the baseline condition, the exposure to prismatic visual displacement requires them to “explore” new strategies (e.g., recalibrate direction or velocity of reaching movement) in order to return to a desired state of accuracy.

Finally, in addition to th LC-NE theory, a number of other neural regions have been implicated as sources of the P300 component. Among these are medial temporal lobe, pre frontal cortex, thalamus, superior temporal gyrus, and inferior parietal lobule (see Linden, 2005 for a review). Generators of the P300 are far less consistent than the ERN, and also suggest, unlike the ERN, the P300 component may result from multiple neural generators.

### Recalibration and realignment

The identification of neural potentials associated with PA is an important development in understanding how visuomotor adaptation takes place during PA. The theory of PA proposed by Redding and Wallace suggests that two processes result in compensation for the prismatic displacement: strategic recalibration and spatial realignment (Redding and Wallace, 1997, 2002, 2006; Redding et al., 2005). Whether the P300 and ERN index neural processes that engage either strategic recalibration or spatial realignment is unknown at this point and further studies will be needed to elucidate this relationship. Given the ERN is evoked solely by miss-trials, it may signal a need for immediate amendments to strategy—thus encourage a process of strategic recalibration on a trial-to-trial basis. On the other hand, because the P300 is sensitive to both hits and misses, but decreases over time, it may signal a slower gradual adjustment to the new visual environment—perhaps akin to spatial realignment.

There is evidence that stroke patients undergo neural changes in visuomotor behavior as a result of PA. For example, Saj et al. (2013) reported increased activation in fronto-parietal brain regions among neglect patients during the performance of visuomotor tasks after a PA procedure, compared to before. Luauté et al. (2006) also reported increased activation in a number of brain regions following PA when compared to before the procedure: the cerebellum, thalamus, temporal/occipital cortex, and posterior parietal cortex. Thus, whether or not they signal recalibration or realignment, the ERPs measured in the present study may index critical neural events that engage changes in how the brain responds to visual stimuli with movement.

### Limitations, future directions

The study was not without limitations. Improvements in methodology should be made to determine the true onset of feedback of accuracy. ERP latencies in the present study are somewhat early given their originally surmised evoking stimulus (feedback of reaching limb). Better control of visual feedback may provide more accurate latencies. Future studies should capture the reaching limb's trajectory and precisely identify when it becomes visible to the participant.

Furthermore, as can be the case with ERP studies, power was an issue in conducting what would have been some appropriate analyses on ERPs. Specifically, it would be informative to measure the effect of various error sizes (not just hit vs. miss) across the three different phases. Unfortunately, there was insufficient power to conduct this analysis. As a result, it is not clear, for example, whether the P300 decreases because overall errors are getting smaller across phase, or simply because the component attenuates over time. Seeing as it is important to utilize repeated-measures design for ERP research, future studies should increase the number of blocks as to increase power and enable more detailed analyses.

Another limitation of the study was that aftereffects were measured solely by magnitude of error at the onset of sham blocks following PA. Although errors during the sham blocks certainly indicated the presence of after-effects, future studies should employ a more standardized measure of after-effect such as a Proprioceptive Visual Straight Ahead (PVSA) task (Redding and Wallace, 2002, 2006; Redding et al., 2005) immediately following PA blocks to better determine magnitude of aftereffects and spatial realignment. This technique would enable us to draw more sound connections between ERP amplitude and degree of spatial realignment/aftereffects.

## Conclusions

This study identified two neural signals, the ERN and P300, which appear to be associated with changes in motor responses to visual targets during prism adaptation. The rapid improvement in error size and MT that occur in conjunction with the ERP responses suggests they play some role in engaging visuomotor adaptation. Extending this research may indeed identify either neural event as critical in modulating after-effects. Not only does this enhance knowledge of neuro-cognitive processes underlying PA, but variance in ERP responses may also help account for individual differences in effectiveness of PA for VSN symptoms.

Individuals with neglect often show difficulty in executing successful movements in response to visual stimuli, but seem to experience respite after undergoing adaptation to prism goggles (e.g., Striemer and Danckert, 2010; Fortis et al., 2011). Therefore, the ERN and P300 may provide key signals to understanding the brain-behavior mechanism that may yield improvements in VSN symptoms. A critical next step in this research is to measure these components in a population of individuals with neglect to determine the role of feedback and error processing in their successful PA treatment.

### Conflict of interest statement

The authors declare that the research was conducted in the absence of any commercial or financial relationships that could be construed as a potential conflict of interest.

## Supplementary material

Figure S1 **Error size across all trials for the four prism exposure conditions: Right 5°, Right 15°, Left 5°, Left 15°**. Errors to the right of the target are recorded as positive values; errors to the left of the target are recorded as negative values.
