# Supplementary figures and images for: Using brain potentials to understand prism adaptation: the error-related negativity and the P300
Source: Front Hum Neurosci. 2015 Jun 12;9:335. doi: 10.3389/fnhum.2015.00335 (PMC4464183; doi:10.3389/fnhum.2015.00335)

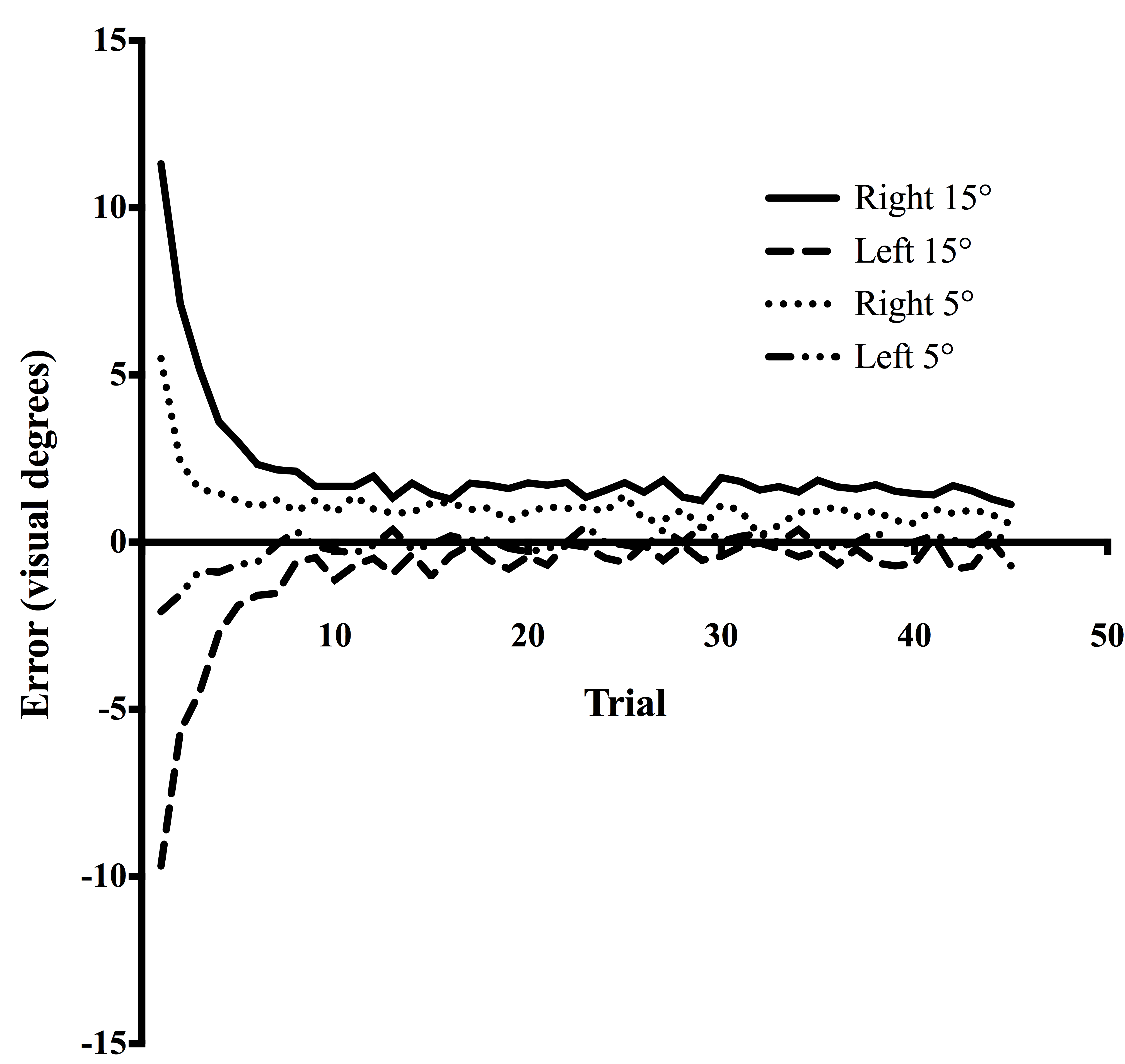

Supplement: Figure S1 — Error size across all trials for the four prism exposure conditions: Right 5°, Right 15°, Left 5°, Left 15°. Errors to the right of the target are recorded as positive values; errors to the left of the target are recorded as negative values. [file Image1.TIFF]
